# Pregnancy Patterns Impact Live Birth Rate for Patients With Intrauterine Adhesions After Hysteroscopic Adhesiolysis: A Retrospective Cohort Study

**DOI:** 10.3389/fphys.2022.822845

**Published:** 2022-03-11

**Authors:** Dan Sun, Xuetao Mao, Aiqian Zhang, Bingsi Gao, Huan Huang, Arvind Burjoo, Dabao Xu, Xingping Zhao

**Affiliations:** ^1^Department of Gynecology, Third Xiangya Hospital of Central South University, Changsha, China; ^2^Department of Obstetrics and Gynaecology, Bruno Cheong Hospital, Central Flacq, Mauritius

**Keywords:** prognosis, live birth rate, hysteroscopic adhesiolysis, intrauterine adhesion (IUA), assisted reproductive technology (ART)

## Abstract

**Objective:**

The pregnancy patterns and other factors of live birth for patients with intrauterine adhesions (IUAs) were identified by analyzing the clinical features of pre-, intra-, and post-hysteroscopic adhesiolysis (HA).

**Design:**

A total of 742 patients with IUAs who wanted to become pregnant underwent HA from January 2017 to May 2018 at the Third Xiangya Hospital of Central South University. The patient follow-up period was 2 years post-HA. A logistic regression was performed to analyze the clinical characteristics associated with a live birth for patients with IUAs. Pre-operative clinical indicators included age, gravidity, parity, abortion, IUA recurrence, menstrual patterns, disease course. Intraoperative clinical features assessed in the last operation were uterine cavity length, IUA appearance, IUA area, number of visible uterine cornua, number of visible tubal ostia, AFS scores. Pregnancy patterns were post-hysteroscopic adhesiolysis features.

**Results:**

Among the 742 IUA patients, 348 (46.9%) had a live birth and 394 (53.1%) did not. A bivariate and binary logistic regression analysis showed that IUA patients’ pregnancy patterns, age, number of visible tubal ostia noted by a second-look hysteroscopy, and American Fertility Society (AFS) scores were significantly related to the live birth rate (*P* < 0.05).

**Conclusions:**

Pregnancy patterns, age, number of visible tubal ostia, and AFS scores were significantly related to the live birth rate and may be considered potential predictors of the live birth rate in IUA patients. The indications of assisted reproductive technology (ART) might be a better choice for patients with recurrent IUAs.

## Introduction

Intrauterine adhesions (lUAs) are acquired uterine conditions that occur when scar tissue forms inside the uterine cavity or cervical canal, causing damage to the basal layer of the endometrium (Asherman, 1948; Sugimoto, 1978). The prevalence of IUAs is difficult to measure, but the number of reported cases appears to have increased over the last few decades.

Intrauterine adhesions occur after trauma to the endometrial lining triggers a wound-healing process that causes the damaged areas to fuse (Kou et al., 2020). Postpartum curettage and surgical abortions are considered major causes of IUAs, and the occurrence rate of severe IUAs in second trimester abortions (69.6%) has been found to be significantly higher than in first trimester abortions (36.7%) (Zhu et al., 2019). Other types of iatrogenic endometrial trauma may be attributed to dilation, curettage, and hysteroscopy (Salazar et al., 2017). Adhesion tissues can partially or completely damage the normal shape of the uterine cavity, leading to a smaller uterine cavity volume, missing tubal ostia, missing uterine cornua, or a loss of the endometrium. Adhesion tissues can seriously impact blood supply to the endometrium, resulting in a thinner endometrium, sparse glandular openings, and decreased endometrial receptivity. Patients with IUAs typically present with amenorrhea, hypomenorrhea, infertility, and repeat abortions (Salma et al., 2014; Chen et al., 2017).

Hysteroscopic adhesiolysis (HA) is the primary treatment for IUAs. It is a procedure that can separate adhesions, restore normal shape to the uterine cavity, improve endometrial blood supply, and make the uterine cavity more suitable for pregnancy (Bosteels et al., 2017; Guo et al., 2019). An intrauterine device (IUD), a Foley catheter balloon, or hyaluronic acid gel is often used as an insertable post-hysteroscopic device to prevent the reformation of adhesions (Hooker et al., 2017). The use of hormones, cell therapies, or amnion membrane grafts could promote the regeneration of the endometrium and aid in the healing process of injured surfaces (AAGL Elevating Gynecologic Surgery, 2017). Despite the available treatments, the prognosis of moderate to severe IUAs is still unsatisfactory. A study found that AFS score has predictive value for post-operative live birth in patients with IUA (Cao et al., 2021). For patients with moderate adhesion, the lower the AFS score, the higher the post-operative pregnancy rate (Zhang et al., 2021b).

So far, there have been many discussions on the etiology and treatment of IUA, but doctors often face the question of whether patients should conceive spontaneous pregnancy or ART after HA. For IUA patients with fertility requirements, under what circumstances it is recommended that patients conceive spontaneous pregnancy after operation, and under which circumstances it is recommended to choose ART directly, there is no research report at present. In this study, we focused on the follow-up of pregnancy outcomes in people who chose spontaneous pregnancy or ART after hysteroscopic adhesiolysis. Pregnancy patterns were used as the main variable for analysis in order to provide patients with more valuable guidance on the optimal patterns of pregnancy after surgery.

## Materials and Methods

### Patients

In this study, 742 patients with IUAs who wanted to become pregnant were enrolled in this study, with all patients having undergone hysteroscopic adhesiolysis at the Third Xiangya Hospital of Central South University from January 2017 to May 2018. Written informed consent was obtained from all patients, and this study was approved by the Ethics Committee of the Third Xiangya Hospital of Central South University. IUAs were scored by the same surgeon who applied the American Fertility Society (AFS) classification system (Valle and Sciarra, 1988). IUAs were scored as follows: 1–4, mild; 5–8, moderate; and 9–12, severe.

The inclusion criteria for this study were as follows: (1) IUAs confirmed by hysteroscopy, (2) a desire to become pregnant, and (3) normal hormone levels and ovulation. The exclusion criteria were as follows: (1) male infertility; (2) primary infertility; (3) tubercular IUAs; (4) tubal factor infertility; (5) other lesions, including endometrial polyps, atypical hyperplasia, or endometrial malignancy; and (6) loss to follow-up.

The clinical features of pre-, intra-, and post-hysteroscopic adhesiolysis were analyzed. Pre-operative clinical indicators included age, gravidity, parity, abortion, IUA recurrence, menstrual patterns, disease course. Intraoperative clinical features assessed in the last operation were uterine cavity length, IUA appearance, IUA area, number of visible uterine cornua, number of visible tubal ostia, AFS scores. Pregnancy patterns were post-hysteroscopic adhesiolysis features. The patients were divided into the live birth group and non–live birth (abortion, stillbirth, or infertility) group. Pregnancy patterns were main variables, including spontaneous pregnancy or ART. The disease course referred to the time interval from the first occurrence of hypomenorrhea or amenorrhea to the first HA. After hysteroscopic adhesiolysis, patients were followed up by telephone for 2 years. The medical records, operative reports, and hysteroscopy videos of these patients were reviewed.

### Surgical Procedure and Post-operative Follow-Up Hysteroscopy

Hysteroscopic adhesiolysis and post-operative follow-up hysteroscopy were performed within 3–7 days following menstruation. Hysteroscopy was carried out using an operative hysteroscope with an outer sheath diameter of 5.4 mm and a working channel diameter of 1.67 mm (Karl Storz SE & Co. KG. KG-Tuttlingen, Baden-Württemberg, Germany).

Pre-operative cervical preparations were not routine. The hysteroscope went through the cervical canal to the intrauterine cavity, and the adhesions were separated gradually from the central part to the lateral wall. As locating the bilateral fallopian tube ostia is crucial to clarifying the anatomy of the uterine cavity (Zhao et al., 2020a), a blunt spreading dissection technique (Zhang et al., 2015) combined with transabdominal ultrasound monitoring was used to increase the visibility of the surgical site. When the intrauterine anatomy was clarified, the IUAs were treated with the cold scissors plowing technique (Zhao et al., 2020b) to reconstruct the normal uterine cavity with clearly visible bilateral fallopian tube ostia. The uterine depth was measured at the end of the operation.

**FIGURE 1:**
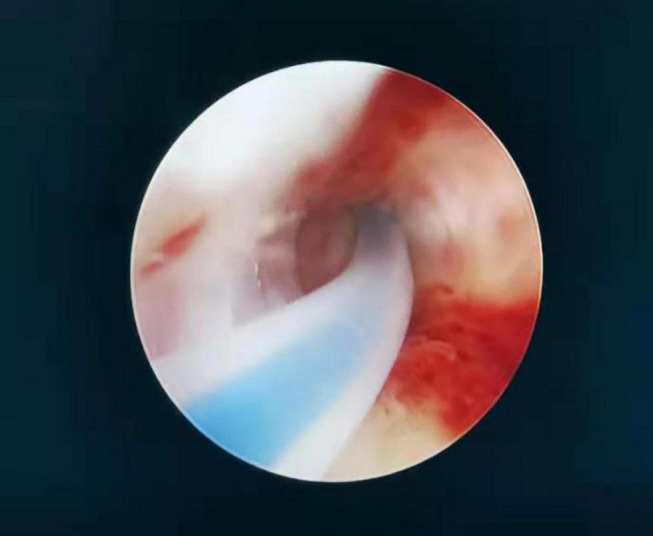
A catheter was inserted through the operation channel of the hysteroscope into the fallopian tube ostia under the guidance of the hysteroscope. Care was taken to maintain a space between the catheter and the fallopian tube ostia so that the catheter could be inserted in the correct direction. The operator fixed the catheter, and the diluted methylene blue solution was injected with a 20-mL syringe by the surgical assistant.

For patients who wished to become pregnant, bilateral intubation of the fallopian tubes under hysteroscopy was completed in the last part of operation (Figure 1). The surgeon inserted the catheter through the operation channel of the hysteroscope into the fallopian tube ostia under the guidance of the hysteroscope. A 0.9% solution of methylene blue in normal saline was injected with a 20 mL syringe, and the resistance, leakage, and backflow of the methylene blue solution was recorded. Reexamination by B-mode ultrasound was performed on the same day as the operation to observe the hydrosalpinx. The patency degree of the fallopian tubes was comprehensively judged according to the above indicators.

After the HA, a uterine-shaped stainless steel IUD was inserted into the uterine cavity, and a double-channel, 12-Fr Foley catheter balloon, with the top catheter portion beyond the balloon removed, was inserted into the uterine cavity. The catheter balloon was then distended using 2.5–3.5 mL of sterile saline according to the size of the uterine cavity, with the balloon positioned in the center of the uterine-shaped IUD after the hysteroscopic adhesiolysis (Zhao et al., 2021). Hyaluronic acid gel (3 mL) was then injected into the uterine cavity *via* the catheter (Pabuqcu et al., 2019).

The hysteroscopic follow-up strategy was developed at the time of the first intervention depending on the AFS score. Further surgery was considered 1 month after the initial operation for patients with severe IUAs to minimize IUA recurrence and 3 months after the initial operation for patients with moderate IUAs to allow the endometrium additional time to heal. Video recordings were made of the patients’ hysteroscopies.

### Statistical Analysis

Statistical analysis was performed using the Statistical Analysis System 9.4 (SAS Institute, Cary NC, United States). The differences between the live birth and the non–live birth groups were tested using a chi-squared test or Fisher’s exact test, as appropriate. A logistic regression analysis was applied to determine the dominant variables for establishing the live birth rate prediction models. The main variable was pregnancy patterns (spontaneous pregnancy and ART), and other clinical characteristics (age, gravidity, parity, abortion, IUA recurrence, menstrual patterns, disease course, uterine cavity length, IUA appearance, IUA area, number of visible uterine cornua, number of visible tubal ostia, and AFS scores) were covariables. A *P*-value < 0.05 was considered statistically significant.

## Results

There were 348 live births (46.9%) and 394 non–live births (53.1%) among the 742 IUA patients. Of the 394 non–live births, 7 experienced late spontaneous abortions between the third and sixth months of pregnancy, while other spontaneous abortions occurred within the first trimester of pregnancy. Pregnancy patterns, age, recurrence of IUAs, menstrual patterns, second-look hysteroscopy uterine cavity length, IUA appearance, IUA area, number of visible tubal ostia, number of visible uterine cornua, and AFS scores were shown to be significantly correlated with pregnancy outcomes (*P* < 0.05). Other variables showed no statistical differences (*P* > 0.05; Table 1).

**TABLE 1:** Related factors affecting live birth rate of fertility-desiring women with IUAs.

Variate	Category	Live birth (%)	Non–live birth (%)	*P*-value
Pregnancy patterns	Spontaneous pregnancy	261 (75.0%)	342 (86.8%)	*P* = 0.0000
	ART	87 (25.0%)	52 (13.2%)	
	Total	348 (100.0%)	394 (100.0%)	
Age	N (NMISS)	348 (0)	394 (0)	*P* = 0.0000
	Mean (SD)	30.4 (4.35)	32.0 (4.89)	
	Median	30	32	
	Q1,Q3	27.0,34.0	28.0,36.0	
	Min–Max	19–41	18–46	
Gravidity	N (NMISS)	3 (0.9%)	3 (0.8%)	*P* = 0.1501
	1	72 (20.7%)	90 (22.8%)	
	2	112 (32.2%)	86 (21.8%)	
	3	66 (19.0%)	84 (21.3%)	
	≥4	95 (27.3%)	131 (33.2%)	
	Total	348 (100.0%)	394 (100.0%)	
Parity	N (NMISS)	4 (1.1%)	3 (0.8%)	*P* = 0.1565
	1	337 (96.8%)	376 (95.4%)	
	2	5 (1.4%)	13 (3.3%)	
	3	0 (0.0%)	2 (0.5%)	
	≥4	2 (0.6%)	0 (0.0%)	
	Total	348 (100.0%)	394 (100.0%)	
Abortion	N (NMISS)	3 (0.9%)	3 (0.8%)	*P* = 0.2899
	1	120 (34.5%)	134 (34.0%)	
	2	109 (31.3%)	105 (26.6%)	
	3	57 (16.4%)	68 (17.3%)	
	≥4	59 (17.0%)	84 (21.3%)	
	Total	348 (100.0%)	394 (100.0%)	
IUA recurrence	Yes	132 (37.9%)	187 (47.5%)	*P* = 0.0088
	No	216 (62.1%)	207 (52.5%)	
	Total	348 (100.0%)	394 (100.0%)	
Menstrual patterns	N (NMISS)	1 (0.3%)	1 (0.3%)	*P* = 0.0037
	Normal	32 (9.2%)	26 (6.6%)	
	Hypomenorrhea	299 (85.9%)	326 (82.7%)	
	Amenorrhea	16 (4.6%)	41 (10.4%)	
	Total	348 (100.0%)	394 (100.0%)	
Disease course	N (NMISS)	36 (10.3%)	43 (10.9%)	*P* = 0.7689
	≤6 months	98 (28.2%)	114 (28.9%)	
	>6 months	214 (61.5%)	237 (60.2%)	
	Total	348 (100.0%)	394 (100.0%)	
Uterine cavity length	N (NMISS)	344 (4)	389 (5)	*P* = 0.0138
	Mean (SD)	7.2 (0.56)	7.1 (0.71)	
	Median	7	7	
	Q1,Q3	7.0,7.5	7.0,7.5	
	Min–Max	6–10	5–9	
IUA appearance	N (NMISS)	4 (1.1%)	1 (0.3%)	*P* = 0.0000
	Filmy	251 (72.1%)	213 (54.1%)	
	Dense	77 (22.1%)	110 (27.9%)	
	Cohesive	16 (4.6%)	70 (17.8%)	
	Total	348 (100.0%)	394 (100.0%)	
IUA area	N (NMISS)	3 (0.9%)	2 (0.5%)	*P* = 0.0000
	≤1/3	332 (95.4%)	333 (84.5%)	
	1/3–2/3	12 (3.4%)	40 (10.2%)	
	>2/3	1 (0.3%)	19 (4.8%)	
	Total	348 (100.0%)	394 (100.0%)	
Number of visible uterine cornua	0	8 (2.3%)	28 (7.1%)	*P* = 0.0003
	1	4 (1.1%)	12 (3.0%)	
	2	336 (96.6%)	354 (89.8%)	
	Total	348 (100.0%)	394 (100.0%)	
Number of visible tubal ostia	0	12 (3.4%)	46 (11.7%)	*P* = 0.0000
	1	10 (2.9%)	40 (10.2%)	
	2	326 (93.7%)	308 (78.2%)	
	Total	348 (100.0%)	394 (100.0%)	
AFS scores	N (NMISS)	347 (1)	394 (0)	*P* = 0.0000
	Mean (SD)	2.7 (1.21)	3.6 (2.15)	
	Median	2	3	
	Q1,Q3	2.0,3.0	2.0,4.0	
	Min–Max	2–10	2–12	

*ART, assisted reproductive technology; IUAs, intrauterine adhesions; AFS, American Fertility Society.*

Univariate logistic regression and multivariate logistic regression analyses both illustrated that different pregnancy patterns affect pregnancy outcomes. Univariate logistic regression showed that the ART patients had a higher live birth rate compared with the spontaneous pregnancy patients (*P* < 0.0001; odds ratio [OR] = 0.456; 95% CI 0.312–0.667; Table 2). ART was also significantly related to better live birth rates in IUA patients according to multivariate logistic regression analysis (*P* < 0.05; Tables 3, 4).

**TABLE 2:** Univariate analysis of the live birth and non–live birth groups.

Variables	Category	Estimate	SE	χ^2^*	*P*-value	Odds ratio	95% confidence interval
Pregnancy patterns	Spontaneous pregnancy	Reference					
	ART	–0.7849	0.1936	16.439	<0.0001	0.456	0.312–0.667
Age		0.0729	0.0162	20.2416	<0.0001	1.076	1.042–1.11
Gravidity	1	Reference					
	2	–0.4873	0.2134	5.2124	0.0224	0.614	0.404–0.933
	3	0.018	0.2282	0.0062	0.9371	1.018	0.651–1.592
	4	0.0982	0.2077	0.2233	0.6365	1.103	0.734–1.658
Abortion	1	Reference					
	2	–0.1477	0.1857	0.6327	0.4264	0.863	0.599–1.241
	3	0.0661	0.2192	0.091	0.763	1.068	0.695–1.642
	4	0.2429	0.2113	1.3217	0.2503	1.275	0.843–1.929
IUA recurrence	Yes	Reference					
	No	–0.3909	0.1496	6.8253	0.009	0.676	0.505–0.907
Menstrual patterns	Normal	Reference					
	Hypomenorrhea	0.2941	0.2759	1.1362	0.2865	1.342	0.781–2.304
	Amenorrhea	1.1486	0.3957	8.4248	0.0037	3.154	1.452–6.85
Disease course	≤6 months	Reference					
	>6 months	–0.0491	0.1669	0.0866	0.7686	0.952	0.686–1.321
Uterine cavity length		–0.3049	0.1177	6.7178	0.0095	0.737	0.585–0.928
IUA appearance	Filmy	Reference					
	Dense	0.5208	0.1754	8.8198	0.003	1.683	1.194–2.374
	Cohesive	1.64	0.2923	31.4718	<0.0001	5.155	2.907–9.143
IUA area	≤1/3	Reference					
	1/3–2/3	1.201	0.3382	12.6134	0.0004	3.323	1.713–6.448
	> 2/3	2.9399	1.0282	8.1753	0.0042	18.915	2.521–141.912
Number of visible uterine cornua	0	Reference					
	1	–0.1541	0.7029	0.0481	0.8264	0.857	0.216–3.399
	2	–1.2006	0.4081	8.6561	0.0033	0.301	0.135–0.67
Number of visible tubal ostia	0	Reference					
	1	0.0426	0.4797	0.0079	0.9293	1.043	0.408–2.672
	2	–1.4005	0.3337	17.6095	<0.0001	0.246	0.128–0.474
AFS scores		0.3378	0.0538	39.4756	<0.0001	1.402	1.262–1.558

*Chi-square test for entire group. ART, assisted reproductive technology; IUAs, intrauterine adhesions; AFS, American Fertility Society.

**TABLE 3:** Full logistic regression of significant variables in the live birth and non–live birth groups.

Variables	Category	Estimate	SE χ^2^*		*P*-value	Odds ratio	95% confidence interval
Intercept		–2.2055	0.74	8.8838	0.0029	/	/
Pregnancy patterns	ART	–0.7084	0.2047	11.9699	0.0005	0.492	0.33–0.736
Age		0.0817	0.0185	19.5597	<0.0001	1.085	1.047–1.125
Gravidity	2	–0.5395	0.2282	5.5905	0.0181	0.583	0.373–0.912
	3	–0.2597	0.2466	1.1099	0.2921	0.771	0.476–1.25
	4	–0.2567	0.2324	1.2204	0.2693	0.774	0.491–1.22
Number of visible tubal ostia	1	0.1427	0.5223	0.0746	0.7847	1.153	0.414–3.21
	2	–0.8108	0.3842	4.4533	0.0348	0.444	0.209–0.944
AFS scores		0.2932	0.0603	23.6501	<0.0001	1.341	1.191–1.509

*Chi-squared test for entire group; ART, assisted reproductive technology; AFS, American Fertility Society.

**TABLE 4:** Stepwise logistic regression of the significant variables in the live birth and non–live birth groups.

Variables	Category	Estimate	SE χ^2^*		*P*-value	Odds ratio	95% confidence interval
Intercept		–2.1892	0.733	8.9207	0.0028	/	/
Pregnancy patterns	ART	–0.6823	0.2038	11.2029	0.0008	0.505	0.339–0.754
Age		0.0802	0.0174	21.1525	<0.0001	1.084	1.047–1.121
Number of visible tubal ostia	1	–0.209	0.5548	0.142	0.7063	0.811	0.274–2.407
	2	–1.0927	0.4242	6.6348	0.01	0.335	0.146–0.77
AFS scores		0.3038	0.0619	24.061	<0.0001	1.355	1.2–1.53

*Chi-squared test for entire group. ART, assisted reproductive technology; AFS, American Fertility Society.

Univariate logistic regression also revealed that the older the patient was, the lower the live birth rate (*P* < 0.0001; OR = 1.076, 95% CI 1.042–1.11). Patients in the non–live birth group were more likely to have the following: (1) recurrent IUAs (*P* = 0.009; OR = 0.676, 95% CI 0.505–0.907), (2) amenorrhea (*P* = 0.0037; OR = 3.154, 95% CI 1.452–6.85), (3) more densely collected adhesions (*P* = 0.003; OR = 1.683, 95% CI 1.194–2.374), (4) more cohesive adhesions (*P* < 0.0001; OR = 5.155, 95% CI 2.907–9.143), (5) larger IUA areas (1/3–2/3: OR = 3.323, 95% CI 1.713–6.448, *P* = 0.0004; >2/3: OR = 18.915, 95% CI 2.521–141.912, *P* = 0.0042), (6) a smaller uterine depth (*P* = 0.0095; OR = 0.737, 95% CI 0.585–0.928), (7) bilaterally invisible uterine cornua (*P* = 0.0033; OR = 0.301, 95% CI 0.135–0.67), and (8) bilaterally invisible fallopian tube ostia (*P* < 0.0001; OR = 0.246, 95% CI 0.128–0.474). Other variables showed no differences between the two groups (*P* > 0.05).

**FIGURE 2:**
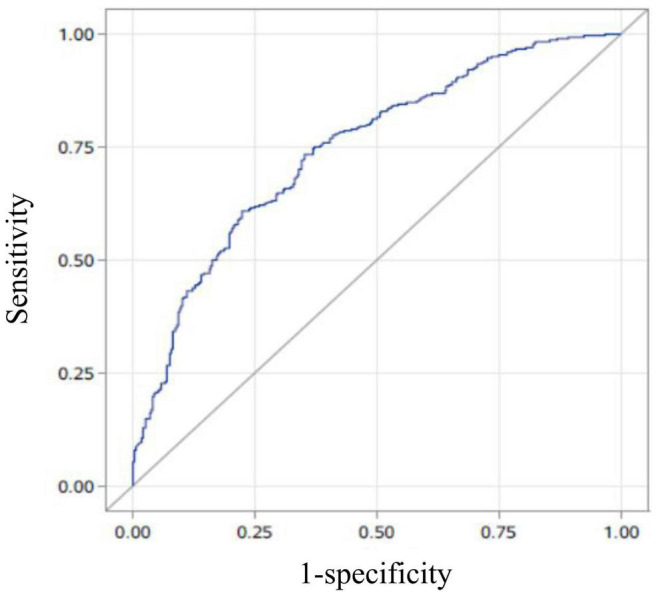
The area under the curve (AUC) of the prediction model. The AUC of the live-birth prediction model (based on pregnancy patterns, age, number of visible tubal ostia, and the American Fertility Society scores in second-look hysteroscopy) was 0.7456.

A multivariate logistic regression analysis was then carried out based on the significant variables (*P* < 0.05) from the univariate logistic regression analysis. Full and stepwise regression methods were used to select the model variables (Tables 3, 4). Pregnancy patterns, age, number of visible tubal ostia, and AFS scores from second-look hysteroscopy were significantly related to the live birth rate of IUA patients (*P* < 0.05), and the model could accurately predict the likelihood of a live birth or a non–live birth (area under the curve [AUC] = 0.7456; Figure 2).

## Discussion

As the incidence of IUAs increases each year, IUAs have become one of the main diseases seriously impacting the reproductive health of women. To date, there have been no reports on the relationship between the choice of pregnancy pattern and pregnancy outcomes after HA. In the interests of maximizing physical and mental health outcomes for patients and minimizing economic costs, it is desirable to establish the most suitable way to approach conception to achieve a live birth.

For women who desire spontaneous pregnancy without ART but who exhibit risk factors affecting pregnancy, such as endometriosis and endocrine abnormalities, successful pregnancy may not be possible shortly after HA, and they may experience recurrence of IUAs in preparing for pregnancy. One study showed that 76% of IUA patients will likely develop new adhesions to differing degrees following their operation (Yang et al., 2013), and our research found that recurrent IUAs were an independent risk factor for live birth (*P* = 0.009). The treatment of recurrent IUA patients is more difficult than that of non-recurrent IUA patients because the fibrotic tissues seriously retard the repair of the endometrium (Lebovitz and Orvieto, 2014). If a recurrent IUA patient were to relapse after surgery, their condition and prognosis would be worse than those of a non-recurrent IUA patient who would be followed up on time after surgery.

As IUAs can result in infertility and repeated loss of pregnancy, they may lead to a harmful cycle (Salazar et al., 2017). Consequently, some patients choose ART post-HA in order to become pregnant as soon as possible. ART is more suitable for female infertility with fallopian tube obstruction or endometriosis, male infertility, recurrent abortion, and genetic diseases (Inhorn and Patrizio, 2015) since ART can limit the influence of high-risk factors on pregnancy outcomes. In addition, ART can help patients choose the time of conception more actively, potentially enabling patients to become pregnant before the recurrence of IUAs. ART may also help reduce the waiting time between surgery and pregnancy, as Deng et al. (2019) noted that the optimal waiting period for a fresh embryo transfer after hysteroscopic adhesiolysis is 90–180 days. In our study, multivariate analysis showed that the final presence of tubal ostia was an independent risk factor affecting the live birth rate. It is known that bilaterally invisible fallopian tube ostia are a sign of a bilateral fallopian tube obstruction and thus an indicator for ART. Our results were consistent with this finding. Consequently, we suspect that ART might be more suitable for patients who have the potential to experience a recurrence of IUAs in order to become pregnant earlier.

In many published studies, the rate of ART after hysteroscopic management of IUAs ranged from 24 to 35% (Pabuccu et al., 2008). In our study, the data showed a rate of only 18.7% (139/742). During their previous HA, the patients with a desire for spontaneous fertility received bilateral intubation of their fallopian tubes under hysteroscopy. It is possible that this operation could improve the patency of fallopian tubes and be beneficial in obtaining spontaneous pregnancy.

In our study, the univariate and multivariate logistic regression models indicated that the live birth rate decreased with age among the IUA patients post-HA. We considered that is a close relationship between age and fertility, the same is true in patients with IUA. The older a patient is, the worse their ovarian reserve function, and the higher the incidence of chromosomal abnormalities during oocyte maturation (Kawwass and Badell, 2018). After the age of 30, the incidence of uterine fibroids and thyroid dysfunction increases significantly (De La Cruz and Buchanan, 2017; Biondi et al., 2019). Therefore, the incidences of spontaneous abortion, ectopic pregnancy, stillbirth, and fetal chromosomal abnormalities in older pregnant women (≥35 years old) are also increased. The incidences of gestational diabetes mellitus, hypertensive disorder complicating pregnancy, abnormal amniotic fluid volume, placenta previa, postpartum hemorrhage, and multiple pregnancy are also significantly increased (Lean et al., 2017; Luo et al., 2020).

In the diagnostic criteria of the AFS, the AFS classification score includes three essential components: IUA area, IUA appearance, and menstruation (Valle and Sciarra, 1988). Univariate analysis found that an IUA area of more than two-thirds of the uterine cavity, dense adhesions, cohesive adhesions, and amenorrhea were all independent risk factors affecting the live birth rate. The multivariate analysis performed in our study showed that final AFS scores were an independent risk factor affecting the live birth rate, and the results were consistent with those of the univariate analysis.

Some studies have pointed out that improving the blood flow of the endometrium may be an important measure in improving its receptivity. The rates of embryo implantation and clinical pregnancy in patients with rich endometrial blood flow are significantly higher than those for patients with poor endometrial blood flow (Savaris and Giudice, 2007; Zhang et al., 2021a). Therefore, hypomenorrhea or amenorrhea might be indicative of a thinner and less receptive endometrium. We found that amenorrhea was an independent risk factor for live birth using a univariate logistic regression model, and this finding was consistent with prior studies.

There are three types of adhesions in the AFS scoring classification: filmy, dense, and cohesive adhesions. IUAs are the abnormal migration and proliferation of epithelial cells and stromal cells, while excessive type I collagen deposits in the endometrium lead to an abnormal repair of fibrosis (Zhu et al., 2017). Endometrial fibrosis will lead to changes in uterine cavity morphology, reduced uterine volume, reduced endometrial glands, and decreased endometrial receptivity, which could seriously affect the establishment of early pregnancy (Cousins et al., 2014). Trauma can destroy the basal layer of the endometrium or the myometrium, and activate TGF-β1/Smad pathway and inflammatory factor (Bai et al., 2020; Leung et al., 2021), which leads to an abnormal mother–fetus interface, resulting in placenta previa or placenta accreta (Zhang et al., 2020). These are all serious obstetric complications that could decrease the live birth rate (Eshkoli et al., 2013).

It has been reported that embryos are mainly implanted in the middle or the upper section of the uterine cavity (Eshkoli et al., 2013). The larger the adhesion areas in the uterine cavity are, the smaller the effective endometrial area. A highly reduced endometrium is unfavorable for embryo implantation and development, as the placenta will more likely adhere to the adhesion zone when the IUA area becomes larger. An abnormal mother–fetus interface can increase the probability of obstetric complications, which may reduce the likelihood of live birth.

As with any study, our investigation has limitations. Performing a more detailed analysis of the complications and comorbidities of pregnancy would be a worthy continuation of our study. Furthermore, our study was retrospective in design and did not implement a random control. The conclusions still need to be confirmed by a large sample of prospective randomized controlled studies.

In conclusion, our research provides a means to more accurately predict the live birth rate and provides insight into the choices patients with IUA can make in achieving pregnancy following HA. With certain indications present, ART might be a better choice for patients with recurrent IUAs.

## Data Availability Statement

The raw data supporting the conclusions of this article will be made available by the authors, without undue reservation.

## Ethics Statement

The studies involving human participants were reviewed and approved by Ethics Committee of the Third Xiangya Hospital of Central South University. The patients/participants provided their written informed consent to participate in this study.

## Author Contributions

DX conceived and designed the study with XZ. DS drafted the manuscript and analyzed the data. XM, AZ, and BG handled the pictures and article format. HH and AB reviewed the data. All authors contributed to the article and approved the submitted version.

## Conflict of Interest

The authors declare that the research was conducted in the absence of any commercial or financial relationships that could be construed as a potential conflict of interest.

## Publisher’s Note

All claims expressed in this article are solely those of the authors and do not necessarily represent those of their affiliated organizations, or those of the publisher, the editors and the reviewers. Any product that may be evaluated in this article, or claim that may be made by its manufacturer, is not guaranteed or endorsed by the publisher.
